# Fe-oxide grain coatings support bacterial Fe-reducing metabolisms in 1.7−2.0 km-deep subsurface quartz arenite sandstone reservoirs of the Illinois Basin (USA)

**DOI:** 10.3389/fmicb.2014.00511

**Published:** 2014-09-30

**Authors:** Yiran Dong, Robert A. Sanford, Randall A. Locke, Isaac K. Cann, Roderick I. Mackie, Bruce W. Fouke

**Affiliations:** ^1^Institute for Genomic Biology, University of Illinois at Urbana-ChampaignUrbana, IL, USA; ^2^Department of Geology, University of Illinois at Urbana-ChampaignUrbana, IL, USA; ^3^Energy Biosciences Institute, University of Illinois at Urbana-ChampaignUrbana, IL, USA; ^4^Illinois State Geological Survey, Urbana-ChampaignUrbana, IL, USA; ^5^Department of Animal Sciences, University of Illinois at Urbana-ChampaignUrbana, IL, USA; ^6^Department of Microbiology, University of Illinois at Urbana-ChampaignUrbana, IL, USA

**Keywords:** deep subsurface, bacterial iron reduction, microbial communities, the Illinois Basin, Mt. Simon Sandstone

## Abstract

The Cambrian-age Mt. Simon Sandstone, deeply buried within the Illinois Basin of the midcontinent of North America, contains quartz sand grains ubiquitously encrusted with iron-oxide cements and dissolved ferrous iron in pore-water. Although microbial iron reduction has previously been documented in the deep terrestrial subsurface, the potential for diagenetic mineral cementation to drive microbial activity has not been well studied. In this study, two subsurface formation water samples were collected at 1.72 and 2.02 km, respectively, from the Mt. Simon Sandstone in Decatur, Illinois. Low-diversity microbial communities were detected from both horizons and were dominated by *Halanaerobiales* of Phylum Firmicutes. Iron-reducing enrichment cultures fed with ferric citrate were successfully established using the formation water. Phylogenetic classification identified the enriched species to be related to *Vulcanibacillus* from the 1.72 km depth sample, while *Orenia* dominated the communities at 2.02 km of burial depth. Species-specific quantitative analyses of the enriched organisms in the microbial communities suggest that they are indigenous to the Mt. Simon Sandstone. Optimal iron reduction by the 1.72 km enrichment culture occurred at a temperature of 40°C (range 20–60°C) and a salinity of 25 parts per thousand (range 25–75 ppt). This culture also mediated fermentation and nitrate reduction. In contrast, the 2.02 km enrichment culture exclusively utilized hydrogen and pyruvate as the electron donors for iron reduction, tolerated a wider range of salinities (25–200 ppt), and exhibited only minimal nitrate- and sulfate-reduction. In addition, the 2.02 km depth community actively reduces the more crystalline ferric iron minerals goethite and hematite. The results suggest evolutionary adaptation of the autochthonous microbial communities to the Mt. Simon Sandstone and carries potentially important implications for future utilization of this reservoir for CO_2_ injection.

## Introduction

Deep terrestrial subsurface ecosystems harbor more than 50% of the microbial biomass (Archaea and Bacteria) present on earth (Whitman et al., 1998), and exhibit unique features that promote a better understanding of biodiversity, microbial adaptive evolution, biogeochemistry, physiological limits to life, and potentially analogous habitats for extraterrestrial life (Fredrickson and Balkwill, 2006). Studies using culture-dependent and -independent methods [e.g., phospholipid fatty acid (PLFA) analyses, molecular analyses of nucleic acids and metagenomics] have revealed phylogenetically diversified and distinct deep subsurface microbiota in a broad range of deep subsurface environments (Lovley and Chapelle, 1995; Pedersen, 2000; Fredrickson and Balkwill, 2006; Chivian et al., 2008). Regardless of how the microorganisms arrived in the ecosystem, the current microbial diversity is controlled by environmental factors, such as burial history, interaction/transport via interconnected fractures, geochemistry and available nutrient and energy sources (Sahl et al., 2008; Lin et al., 2011). As physically and chemically isolated ecosystems, deep subsurface environments are often depleted in readily utilizable fixed photosynthetic organic carbon. Instead, a broad range of organic and inorganic nutrients, such as buried kerogen, petroleum components, gasses (e.g., H_2_ and CH_4_) or reduced ions (e.g., sulfur species and different metals) (Lovley and Chapelle, 1995; Liu et al., 1997a; Zhou et al., 2001; Fredrickson and Balkwill, 2006), enable deep subsurface microbes to use various strategies to conserve energy for survival and metabolism. These microbial processes likely occur at extremely low rates in these natural subsurface environments and measuring changes may be difficult over short periods of time (Hoehler and Jorgensen, 2013).

Microbial iron reduction was suggested to be the first external electron acceptor of global significance in microbial metabolism in early Earth (Vargas et al., 1998). Abundant and ubiquitous ferric iron oxides can serve as electron acceptors for dissimilatory iron reduction in a variety of environments (Lovley, 1991; Lovley and Chapelle, 1995). Microbial iron reduction has been identified in virtually all the known deep subsurface ecosystems, including marine and continental petroleum deposits, hydrothermal vents, mines, and deep sedimentary basins (Lovley, 1991; Fredrickson et al., 1998; Pedersen, 2000; Lovley et al., 2004). Since the first discovery of the iron-reducing organisms in two extremely deep subsurface reservoirs, the Triassic-age Taylorsville Basin in Virginia and the Cretaceous-age Piceance Basin in Colorado (Liu et al., 1997a), phylogenetically diversified iron-reducing organisms from bacterial and archaeal domains have been identified in other subsurface habitats. These organisms are capable of using poorly crystalline (e.g., ferrihydrite) and/or well crystalline ferric iron minerals (e.g., goethite, hematite and magnetite) as the terminal electron acceptor, coupled with a variety of organic and inorganic substrates (e.g., H_2_) as electron donors (Lovley, 1991). The iron reducers identified in deep subsurface habitats are typically obligately anaerobic, mesophilic, or thermophilic, capable of tolerating a broad range of temperature and nutritionally versatile. In addition to iron reduction, most of them also exhibit other metabolic capacity, e.g., fermentation, methanogenesis, and reduction of sulfate, nitrate or other metals (Slobodkin, 2005; Fredrickson and Balkwill, 2006).

The Mt. Simon Sandstone is a Cambrian-age sandstone that overlies Precambrian basement of the Illinois Basin (Sloss, 1963; Buschbach, 1964). Geophysical, hydrological and geochemical evidence suggest that this geological formation (Mt. Simon Sandstone) has been isolated from surface processes for millions of years (Swann, 1968; Fishman, 1997; Leetaru and McBride, 2009). The deeply buried Mt. Simon Sandstone contains quartz grains with ubiquitous iron oxide cement coatings and porosity filled with briny water with high concentrations of dissolved ions (Bowen et al., 2010; Locke et al., 2013; Dong et al., 2014).

Previous studies of the biosphere inhabiting the Illinois Basin exhibit that the microbial communities and their metabolic capacities were stratigraphically variable, possibly due to the indigenous burial history and resulting geological, physical, and geochemical environments. The studies on the Pennsylvanian Seelyville coal beds (95–100 m depth) on the eastern margin of the Illinois Basin in Indiana (Strapoć et al., 2009) revealed a microbial community dominated by *Methanocorpusculum*, which may utilize H_2_ and CO_2_ derived from biodegradation of coal organic matter for methane production (Strapoc et al., 2008). In addition, Dong et al. (2014) identified a lowly diversified microbial community inhabiting in the significantly deeper (1.8 km) and more ancient subsurface of the Cambrian-age Mt. Simon Sandstone. This microbial community dominated by *Halomonas* may use/recycle extracellular and intracellular nutrients to survive the anaerobic, warm, saline and oligotrophic environmental stress (Dong et al., 2014).

In this study, microbial communities were sampled and characterized from two horizons (1.72 and 2.02 km depth) within the Mt. Simon Sandstone. Bacterial enrichments were successfully grown from the formation pore water collected from these two horizons. Each bacterial enrichment was characterized for phylogenetic diversity and metabolic activity to assess their relatedness to known taxa and to determine their capacity to survive the indigenous subsurface geochemical environment of the Mt. Simon Sandstone (e.g., tolerance to the simulated indigenous geochemical conditions, utilization of indigenous ferric iron-oxide minerals).

## Geological setting

**Figure 1:**
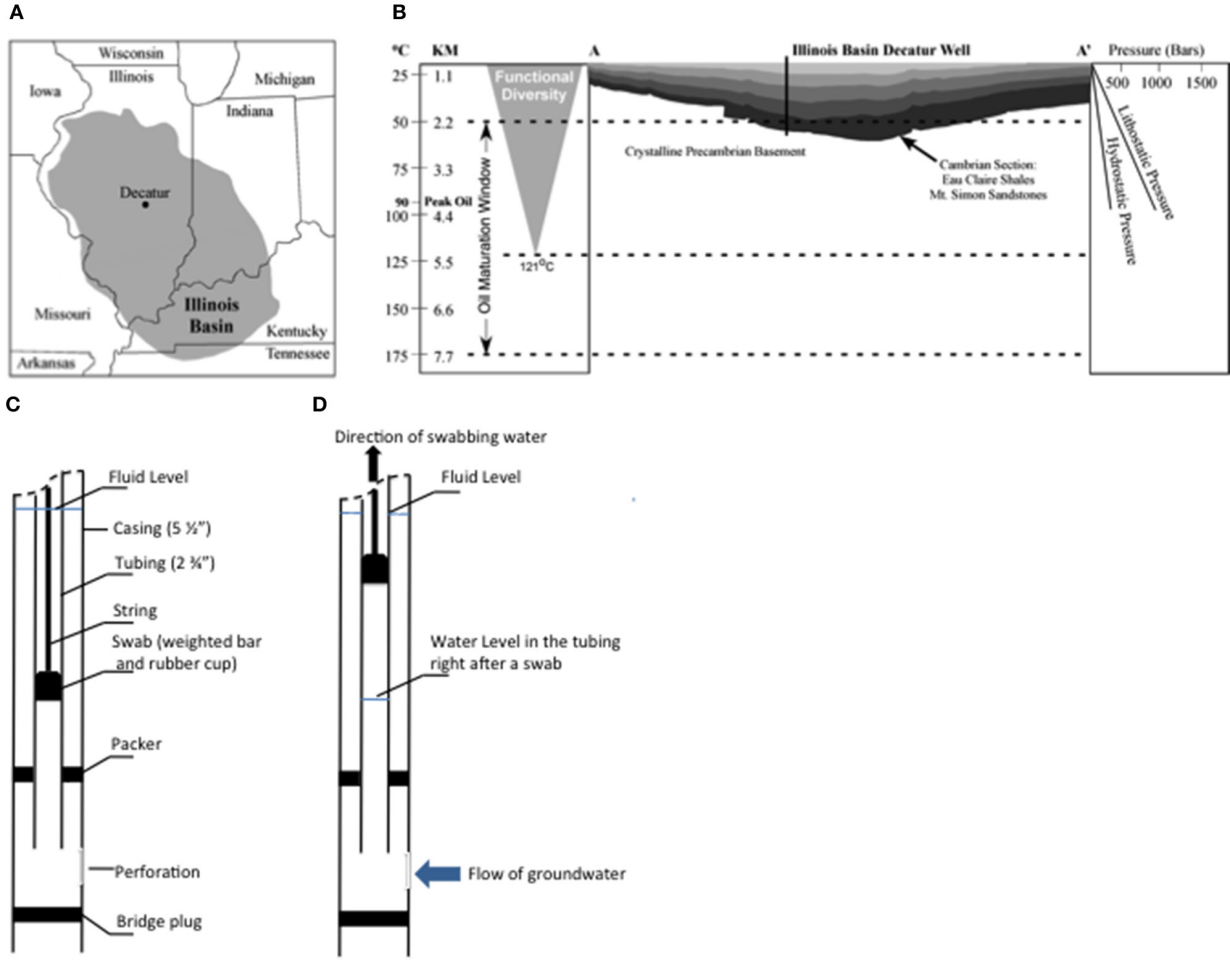
**Map of the Illinois Basin with the area highlighted in gray color **(A)**, the geological line of section A–A' **(B)** and diagrams of swabbing technique (**C,D**)**. In **(B)**, the changes of temperature and pressure along depth of the Illinois Basin are presented on both sides of the diagram.

The Illinois Basin is an oval-shaped depression that covers 284,899 km^2^ of the U.S. Midcontinent (Kolata, 2010) (Figure 1). As depicted by burial curves and thermal history modeling and homogenization temperatures of 100–130°C from fluid inclusion data, the Illinois Basin reached a maximum burial depth of about 2.4 km and temperature of approximately 100°C (Fishman, 1997; Rowan et al., 2002; Makowitz et al., 2006). Multiple episodes of tectonic and thermal subsidence from the late Precambrian and continuing throughout the Phanerozoic (Buschbach, 1964; McBride, 1998) strongly influenced deposition of Paleozoic sequence of terrestrial to marine siliciclastics (primarily sandstones and shales) and marine carbonates within the Illinois Basin. These events also produced well-sealed Paleozoic sandstone, limestone, and shale deposits that form excellent reservoirs for both hydrocarbon entrapment and regional CO_2_ sequestration (Leetaru et al., 2009; U.S. Department of Energy, National Energy Technology Laboratory, 2010).

The Mt. Simon Sandstone is composed of deposits of Cambrian-age at the base of the Sauk Sequence (Sloss, 1963; Buschbach, 1964; Bowen et al., 2010) (Figure 1B). This geological formation (Mt. Simon Sandstone) is the target reservoir of the Illinois Basin—Decatur Project (IBDP), a one million tonne carbon sequestration demonstration project funded by Department of Energy and led by the Midwest Geological Sequestration Consortium. IBDP is the first carbon storage project in North America to store CO_2_ from an biofuel source in a deep saline reservoir (Leetaru et al., 2009). This deposit is overlaid with two major shale layers, including the Eau Claire and New Albany Shales, which provide a natural seal for the CO_2_ sequestration project (Leetaru et al., 2009). The Mt. Simon Sandstone is heterogeneous in composition, consisting of six lithofacies: (1) cobble conglomerate, (2) stratified gravel conglomerate, (3) poorly-sorted sandstone (most common), (4) well-sorted sandstone, (5) interstratified sandstone and shale, and (6) shale (Driese et al., 1981; Fishman, 1997; Leetaru and McBride, 2009; Bowen et al., 2010). The stratigraphic heterogeneity and distribution of different lithofacies is reflective of the transition from alluvial fan and braided river to the marine lagoon depositional environments from deeper to shallower formations of the Mt. Simon Sandstone (Houseknecht and Ethridge, 1978; Leetaru and McBride, 2009; Bowen et al., 2010). The high porosity and permeability of the Mt. Simon Sandstone account for its estimated CO_2_ storage capacity of at least 11–150 billion metric tons within the Illinois Basin (DOE, 2012).

Petrographic and spectral analyses have shown that ferric iron minerals (i.e., hematite and goethite) are common throughout the Mt. Simon Sandstone. This was reflective to the striking color variation and iron oxide bands of the sandstones in this geological formation (Bowen et al., 2010; Dong et al., 2014). These iron oxides exhibited a variety of morphology, including grain coatings or apparent “displacive” dispatches. Whole-rock geochemical analyses indicated that the iron-containing grains typically compose of 1.3–2.1% iron oxides, while the content can be up to 10% in some samples (Bowen et al., 2010).

## Materials and methods

### Sampling

An intensive regime of subsurface fluid sampling was performed using the IBDP verification well (VW1), Decatur, IL (Figure 1), during May 4 to May 18, 2011.

All field sampling was completed in close collaboration with the Illinois State Geological Survey (ISGS) and Schlumberger Carbon Services. After drilling completion and before the well was perforated, VW1 was filled with a synthetic brine solution (the Decatur municipal water mixed with NaCl, density of approximately 1.1 kg/L) to balance the fluid pressure between the well and the formation. The VW1 well casing was then perforated at targeted depth intervals. The day before sampling started, the synthetic brine solution was removed for disposal.

Subsurface formation water was collected from target horizons using the “swabbing” technique (Locke et al., 2013; Panno et al., 2013) (Figures 1C,D), in which formation fluid from different formation depths within packer isolated zones in the well was effectively pumped to the surface using a sealed swab (rubber cup). A tubing string was placed in the well with a removable bridge plug and inflatable packer assembly at the bottom of the string to isolate individual sampling zones. At the top of the tubing string above land surface, a swabbing tool was attached. A swabbing tool string includes an assembly known as the swab, which is comprised of a weighted bar and a rubber swab cup that were lowered into the tubing string below the fluid level about 500 to 1000 feet (Figure 1C). When the swab was retrieved quickly, the swab cups lifted the fluid to the surface where it was discharged at the wellhead (Figure 1D). For each pull of the swab (called a “*swabbing*”), approximately 178–358 liters of fluid was produced. For each swab, about 200 mL fluid was collected for geochemical analyses on site. During swabbing, the pressure decrease in the tubing allowed formation water to enter the tubing, which typically took 0.5–1.5 h to recover the original water level. Then, the rubber swab cup was lowered again and swabbing was repeated (Figures 1C,D) until selected geochemical parameters did not vary significantly with subsequent swabs (i.e., reached stable conditions).

In this study, large volume of formation water was pumped before sample collection to obtain the formation fluid representative of the indigenous deep subsurface environments. Mt. Simon Sandstone is a highly heterogeneous sedimentary rock reservoir, thus water properties can vary naturally due to heterogeneity within this geological formation. Producing water until field parameters stabilize is the attempt to purge non-native fluids and homogenize the fluid sampled so that it is representative. With great volumes of purged water, a larger volume of reservoir is involved to supply the fluid and should minimize atypical chemical environments that would be closer to or immediately adjacent to the well bore. Twelve swabs of formation fluid from Zone 9 and 46 swabs from Zone 6 were pumped out, respectively. A suite of geochemical parameters, including pH, specific conductance, dissolved oxygen, oxidation-reduction potential (Eh) were measured on the produced water from each swab. Other parameters, such as major anions, cations, trace metals, ammonium/ammonia, total dissolved solids, alkalinity, and fluid density were either promptly measured in the field on periodic samples or analyzed in the laboratories of the ISGS Geochemistry Section or Illinois State Water Survey (ISWS) Public Service Laboratory (Supplementary Information S1.1 and S1.2).

### Sampling design to minimize contamination

In the present study, to evaluate potential contamination introduced during sample collection, two samples were collected during the swabbing of each subsurface horizon. The first sample from each depth represented water still contaminated with non-native fluids, and the second sample was the last swab taken after the field parameters stabilized and was the most geochemically representative formation sample. Swabs 6 and 14 were from VW1 Zone 9 at 1.72 km in depth (5655-S6 and 5655-S14, respectively), while Swabs 24 and 46 were collected from the VW1 Zone 6 at 2.02 km (6634-S24 and 6634-S46, respectively). During swabbing, the zones of interest were sealed off with the packer system to isolate and discrete target horizons. Thus, the final formation waters collected after the long-duration (1–2 days) swabbing of large volume of formation fluid should be representative of the deep subsurface formation water conditions. Similar technique has been employed in other studies collecting deep subsurface formation water (e.g., deep aquifers in Montana and Columbia Basin, WA) (Olson et al., 1981; Lavalleur and Colwell, 2013).

### Molecular analysis of microbial communities

Genomic DNA was extracted from the filter membranes, through which freshly collected formation water was filtered as introduced in the Supplementary Information. The conical tubes containing membranes in RNAlater were thawed on ice before centrifuged at 9000 rpm at 4°C for 45 min. The RNAlater supernatant was carefully removed and the filter membranes were blotted on sterilized Kimwipes®. The protocol for DNA extraction was modified from a previously published protocol (Tsai and Olson, 1991). Briefly, a lysis buffer containing pyrophosphate (He et al., 2008) and lysozyme (15 mg/mL) was used to minimize absorption of DNA on native iron minerals. This was followed with another incubation in STS solution (0.1 M NaCl, 0.5 M Tris-Cl, pH = 8.0, 10% sodium dodecyl sulfate) (He et al., 2008) and freeze-thaw cycling between a 55°C water bath and liquid nitrogen to further disrupt cell membranes for three times. After sequential phenol, phenol:chloroform (5:1) and phenol:chloroform:amyl alcohol (25:24:1) extractions, the supernatant was mixed with two volumes of Solution 3 of Ultraclean Soil DNA Isolation Kit (Mobio Laboratories, Inc., CA). The mixed solution was loaded on a silica spin filter and centrifuged at 10,000 × g. The filtrate was discarded and this step was repeated until all the mixed solution was filtered. The following DNA wash and elution was performed following the manufacture's recommendation. Using silica filters to collect DNA circumvented co-precipitation of DNA and salts when classical alcohol precipitation was applied, which might be due to the high salt concentrations in the samples. Unless mentioned, a negative control under the same extraction process was prepared to ensure no contamination was introduced during DNA preparation.

In order to extract DNA from the active enrichment cultures, about 3 mL of well-mixed culture developed from the formation water samples 5655 and 6634 (referred to as IBDP5655 and IBDP6634 afterwards, respectively) was withdrawn with a N_2_ flushed syringe and transferred into microcentrifuge tubes. The samples were centrifuged at 13,000 rpm at 4°C for 10 min and the supernatant was discarded. DNA was extracted from the pellets by using FastDNA® Spin Kit for Soil (MP Biomedicals, CA).

Microbial community composition in the formation water samples and active enrichment cultures were characterized using 16S rRNA clone libraries. Primers used for amplifying 16S rRNA genes were the Bacteria-specific primers 8F and 1492R (Liu et al., 1997b) and Archaea-specific primers 21F and 958R (Elshahed et al., 2004). PCR was conducted with TaKaRa *Ex Taq* polymerase (TaKaRa Bio USA, Madison) using an Eppendorf MasterCycler (Eppendorf, Germany) following the recommendation of the manufacturers. The PCR products were verified by using agarose gel electrophoresis and then purified with QIAquick PCR Purification Kit (QIAGEN Inc., CA). Purified 16S rRNA genes were cloned into p-GEMT Easy Vector® and transformed into JM109 high efficiency competent cells as recommended by the manufacturer (Promega Corporation, WI). For each 16S rRNA clone library, a total of 192 recombinant plasmids were extracted from randomly picked *E. coli* colonies containing cloned sequences. The cloned insert in each plasmid DNA sample was amplified and sequenced using M13 primers at the Illinois Biotechnology Center of University of Illinois Urbana-Champaign, Urbana.

The 16S rRNA gene sequences from the clone libraries were assembled with Sequencher 4.9 (Gene Codes Corporation, Ann Arbor, Michigan). Sequences with low quality (e.g., not able to be assembled, ambiguity > 5 or with trimmed ends) were eliminated. Alignments and distant matrices of assembled sequences were generated with the aid of the NAST package in Greengenes (DeSantis et al., 2006). Chimeric artifacts were determined with Bellerophon (version 3) (Huber et al., 2004) and removed. Operational taxonomic units (OTU) were designated at sequence similarity levels of 97% with Mothur 1.28.0 (Schloss et al., 2009). Taxonomic classification was determined by sequence classification program from the Greengenes (DeSantis et al., 2006) and confirmed with Ribosomal Database Project (RDP) II database (Maidak et al., 2001). A neighbor-joining phylogenetic tree was constructed with the aid of ARB based on Jukes-Cantor correction (Ludwig et al., 2004; Tamura et al., 2007). The robustness of the inferred tree topologies was evaluated after 1000 bootstrap replicates of the neighbor-joining data.

### Enrichment cultures

In order to enhance growth of indigenous microorganisms from VW1, a series of enrichment cultures were established. These were prepared by inoculating formation water collected from different horizons into a synthetic basal medium modified from Roh et al. (2002). The trace metal and vitamin solutions in the original basal medium (Roh et al., 2002) were replaced by 10 mL/L Balch trace metal solution (Dworkin et al., 2006) and 1 mL/L ATCC vitamin solution (American Type Culture Collection, VA). The medium was equilibrated with N_2_/CO_2_ (80:20, v:v) at pH 7.0–7.2. Six mL of basal medium was added into a serum tube, flushed with N_2_/CO_2_ and sealed with a blue butyl rubber stopper and an aluminum seal before sterilization. Degassed formation water samples (4 mL) were added into the serum tubes after bubbling with N_2_ gas. To test the different electron accepting processes, amendments of ferric citrate, sodium sulfate and sodium nitrate (5 mM) were added as electron acceptors to enrich for iron-, sulfate- and nitrate-reducing activities, respectively. Acetate, lactate and pyruvate (5 mM each) and H_2_ (5 mL/tube) were added as electron donors and carbon sources. Separate enrichment culture tubes received 5 mM methanol to evaluate methanogenesis potential or glucose (5 mM) to evaluate fermentation activity. The enrichment cultures were incubated at 42°C. Every 10–20 days, growth of the enriched microorganisms was observed microscopically using a Zeiss Standard 25 microscope (Carl Zeiss Inc., Germany) and activity was monitored by visual observation of color change in the cultures (e.g., microbial iron reduction led to color changes from reddish brown to greenish yellow with ferric citrate as the electron acceptor) or by measuring changes in the concentration of donors or acceptors.

After cells were observed in the enrichment cultures based on microscopic visualization, they were subcultured into fresh media prepared using the same conditions. To better match natural ecosystem conditions, filter sterilized (0.22 μm) formation water was added into the basal medium and accounted for 40% of the volume. A 10% inoculum of the parental enrichment culture was transferred into the subculture using a N_2_ flushed sterile syringe. The subcultures were incubated statically in the dark at 42°C and manually inverted once every day. Enrichment cultures were maintained by transferring into fresh medium about every 4 weeks using a 10% inoculum. The physiological properties of iron-reducing enrichment cultures and the mineral characterization during iron reduction were characterized as described in the Supplementary Information (SI S1.3 and S1.4, respectively).

### Evaluation of contaminant contribution to microbial community

In order to evaluate whether the enriched microorganisms were indigenous to the formation water or introduced populations from the non-native fluids (e.g., drilling fluid), the identified predominant OTUs identified in the enrichment cultures were quantified in both the intermediate partially contaminated swab sample (e.g., 5655-S6 and 6634-S24) and the most representative sample of formation water (e.g., 5655-S14 and 6634-S46). The OTUs were quantified by qPCR analysis using OTU-specific (i.e., species-specific) primers for 16S rRNA genes compared to the total bacterial 16S rRNA genes using universal 16S rRNA gene primers (Ritalahti et al., 2006). OTU-specific primers were designed using Oligo (Molecular Biology Insights, Inc., CO) (Table S1). SYBR *Premix Ex Taq* (Tli RNAseH+) (Takara Bio Inc., Japan) and 200 nM of each primer were combined in sterile, nuclease-free water (Invitrogen) prior to addition of any genomic DNA template. qPCR was performed with a Mx3000P PCR machine (Agilent Technologies, CA). The designed primer targeting the specific regions of pairs both optimally utilized at annealing temperature of 55°C with the PCR cycles parameters as follows: initial denaturation stage of 30 s at 95°C, followed by 40 cycles of PCR 5 s at 95°C, 30 s at 56°C, and 30 s at 72°C and 1 cycle of dissociation or melting curve stage by increasing temperature from 55°C to 95°C at the rate of 0.2°C/s. The universal primers Bac1055YF and Bac1392R (Ritalahti et al., 2006) were used to quantify the total bacterial 16S rRNA genes. qPCR calibration curves were obtained using serial dilutions of plasmids inserted with a single 16S rRNA gene of either the 5655 or 6634 enriched species. The amplification efficiencies were calculated by the Pfaffl Method (Pfaffl, 2001). The targeted gene copy numbers were calculated following the method as described (Ritalahti et al., 2006).

### Data accession

The 16S rRNA gene sequences have been submitted to the National Center for Biotechnology Information (NCBI) GeneBank with the accession numbers KM105781-KM105838.

## Results

### Subsurface formation water geochemistry of the Mt. Simon sandstone

Physical and water chemistry analyses of the samples collected from 1.72 to 2.02 km (5655 and 6634 feet, respectively and thus, referred to as 5655 and 6634 hereafter) indicate both horizons are moderately warm (47–51°C), highly pressured (170–250 bars), weakly acidic (pH 6.31–6.39) and saline (Table 1). Differences for a series of geochemical parameters were observed not only between the intermediate swab samples and their final counterparts but also between the final samples collected from the two horizons. The 6634 samples exhibited a higher salt concentration than the 5655 samples; with roughly 258 and 167 ppt total dissolved solids (TDS), respectively (Table 1). Na^+^, K^+^, Ca^2+^ and Cl^−^ contributed to the majority of salinity in both samples; however, there was variation in salt concentration between the horizons, which may explain the higher density of 6634 (D = 1.1375 g/mL) relative to 5655 (D = 1.0979 g/mL). Aqueous ferrous iron concentrations ranged from 1.18 to 1.37 mM in both water samples. These concentrations were high enough to allow visible evidence of iron oxidation (i.e., samples turned yellowish red) after samples had been exposed to air. For both 6634 and 5655 samples, the concentrations of total organic carbon (TOC) (56.5 and 55.4 mg/L, respectively) and total nitrogen (TN) (19.5 and 12.3 mg/L, respectively) were low. Intermediate samples (5655-S6 and 6634-S24) were lower in density, concentrations of ferrous iron, and TDS than the final samples (5655-S14 and 6634-S46) (Table 1). The formation water from both horizons indicated highly reduced environments with significantly negative electrical potential (about −80 mV). These measurements showed that the water chemistry changed during swabbing and that potential contaminants (e.g., residual synthetic brine solution, drilling fluid) were removed after sufficient purging (i.e., swabbing) was performed.

**Table 1:** **Geochemistry of formation water^a^**.

	**5655 (1.72 km)**		**6634 (2.02 km)**	
	**Intermediate^b^**	**Final^b^**	**Intermediate**	**Final^b^**
Sample Name	5655-S6	5655-S14	6634-S24	6634-S46
Temperature (°C)	−^c^	47	–	50
Pressure (bars)	–	170	–	250
pH^d^	6.27	6.31	7.11	6.40
Electrical conductivity (m/s)	148	151	160	142
Electrical potential (mV)	−73.8	−84.9	−138.3	−84.0
Density (g/mL)	1.0979	1.1009	1.1076	1.1375
Fe(II) (mM)	1.13 ± 0.03	1.18 ± 0.01	0.95 ± 0.07	1.37 ± 0.02
TOC (mg C/L)	143.2 ± 2.7	55.4 ± 1.3	3.33 ± 0.13	56.5 ± 1.2
TDS (g/L)^e^	165 ± 2	167 ± 2.5	169 ± 1	258 ± 7
Ions				
Cl^−^ (mg/L)	–	88236	–	120438
Br^−^(mg/L)	–	536	–	713
SO^2−^_4_(mg/L)	–	931	–	291
Ca^2+^(mg/L)	–	13353	–	21464
K^+^(mg/L)	–	1686	–	2272
Na^+^(mg/L)	–	48896	–	47156
Zn^2+^(mg/L)	–	1.858	–	1.933
Cl/Br	–	164		168
Na/Br	–	91		66
K/Cl	–	0.019		0.019
ALK^f^ (mg/L CaCO_3_)	–	105 ± 19	–	112 ± 22
TN (mg/L)^f^	–	12.3 ± 0.4	–	19.5 ± 1.4

a T, pH, DO and density were immediately determined after sample collection at atmospheric pressure in the field, while other parameters were measured on pre-treated samples in the lab;

b Intermediate samples were collected before field sampling parameters stabilized. Final samples were collected after field sampling parameters stabilized (i.e., from the last swab). The values indicate average of the measurements on replicate samples or in different laboratories; the typical uncertainties are less than 7.5%;

c -: not determined;

d Uncertainty of measuring replicates was typically within ± 0.02.

e TDS and TN were measured as the total solid content in freeze-dried formation water that passed through membrane with 0.22 μm pores.

f ALK indicates alkalinity.

### Molecular analyses of enrichment cultures

Active iron-reducing enrichment cultures were developed in IBDP5655 and IBDP6634. With ferric citrate as the electron acceptor and a mixture of fatty acids [e.g., pyruvate, acetate, lactate, butyrate (5 mM each)] and H_2_ (5 mL/tube) as the electron donors, ferrous iron accumulation was noted within 2 weeks. Faster initial iron reduction was detected in IBDP5655, while slower Fe(II) accumulation occurred with IBDP6634, however this enrichment culture yielded a higher final Fe(II) concentration (10.96 mM) (Figure S1).

The organisms that grew in the enrichment cultures were identified by sequencing 16S rRNA gene clone libraries from DNA amplified from each culture. Results revealed low bacterial diversity but significantly different bacterial populations in the communities between IBDP5655 and IBDP6634. For IBDP5655, two OTUs (97% cutoff) were identified, both of which were affiliated to Class Bacillales and showed about 95% in identity to the16S rRNA gene from *Vulcanibacillus modesticaldus*, an isolate from a deep ocean hypothermal vent (L'Haridon et al., 2006). Despite being only a secondary enrichment culture, IBDP6634 only contained a single OTU, which was affiliated to Class Halanaerobiales. This OTU was about 96% identical to 16S rRNA genes from *Orenia marismortui*, an isolated halophile from the Dead Sea (Moune et al., 2000).

### Evaluation of possible sampling contamination

**Figure 2:**
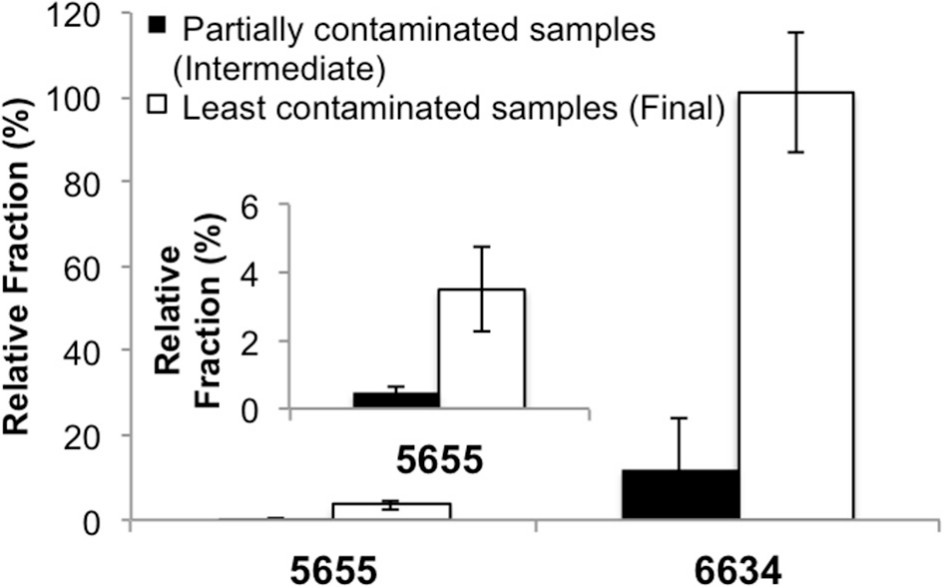
**qPCR quantification of relative fractions of *Vulcanibacillus* sp. and *Orenia* sp. enriched from the formation water bacterial communities**. The partially contaminated samples (Sample 5655-6S and 6634-S42) were collected during “swabbing” before geochemical conditions became stable and contained more contamination, while the least contaminated samples (Sample 5655-S14 and 6634-S46) were the final swabs after the *in-situ* geochemical parameters leveled off. The subfigure indicates the results of 5655 illustrated with narrower scale.

To test that the enriched populations within the cultures were not from the contaminating fluids in the wells, several quality assurance analyses were done. Assuming contaminating microbial populations introduced from residual drilling fluid or other sources would “dilute” indigenous microbial communities, indigenous populations should account for a higher fraction of the community in the “cleaner” samples than in the intermediate samples (Unno et al., 2010, 2012). Quantitative PCR (qPCR) was applied to determine fractions of the enriched species in the microbial communities of the intermediate vs. the final samples (e.g., 5655-S6 vs. 5655-S14, 6634-S24, vs. 6634-S46). Species-specific primers targeting the variable regions of the 16S rRNA genes of the enriched *Vulcanibacillus*- and *Orenia*- related populations were designed (Table S1). Specificity of the primers was verified in the preliminary experiments and both showed a single product in regular PCR and a single denaturing peak in qPCR (data not shown). The species-specific 16S rRNA genes were normalized with the total 16S rRNA gene copy numbers of each corresponding microbial community to quantify the contributing fractions of target populations to whole microbial community. For IBDP5655, the *Vulcanibacillus*-related species was a minor OTU and accounted for less than 5% of this microbial community in abundance, while *Orenia*-related species was a major OTU contributing to 16–100% of IBDP6634 (Figure 2). More importantly, the fractions of the enriched *Vulcanibacillus*-species from IBDP5655 and *Orenia*-related species from IBDP6634 were significantly lower in the partially contaminated intermediate formation water samples than in the final “cleaner” samples. This indicates that the bacterial populations enriched in IBDP5655 and IBDP6634 are indigenous microorganisms in the deep subsurface of the Illinois Basin.

### Molecular analyses of the Mt. Simon formation bacterial communities

**Figure 3:**
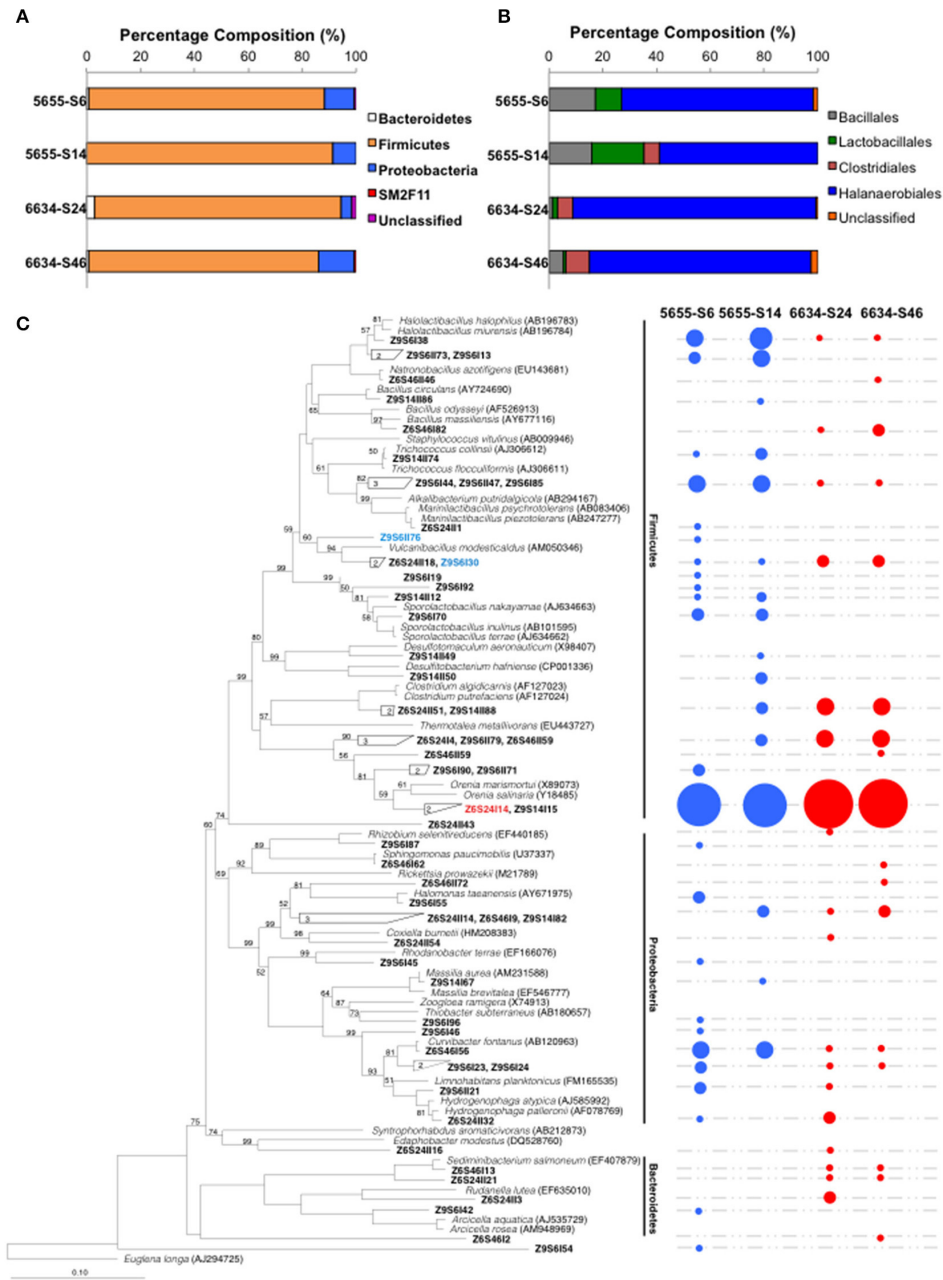
**Microbial composition (A), the dominant Firmicutes phylum (B) and phylogenetic tree (C) of the microbial communities inhabiting in the formation water collected from 1.72 to 2.02 km in depth of the IBDP verification well VW1**. The values n and n' listed on the top of the figures **(A,B)** indicate number of 16S rRNA used for analyses. Both the intermediate and final samples are included in this analysis. The IDs in bold fond in **(C)** indicate the OTUs of the microbial communities identified using 97% as the cutoff value. The OTUs belonging to the same clusters were grouped together and the numbers of grouped OTUs were labeled in the clades. The circles on the right side indicate the presence of detected OTUs and their size is proportional to the fractions of the corresponding OTUs based on the clone library analysis. The species in italic font were type strains selected from RDP database and most closely related to the detected clones. The NCBI accession numbers of the type strains are listed in the parentheses. The scale bar indicates 0.1 changes per nucleotide position. Statistical confidence for the evolutionary tree was assessed by bootstrap (1000 replicates) and shown as bootstrap values in percentage. The organisms that were successfully enriched from IBDP5655 and IBDP6634 are shown in blue and red colors, respectively.

The bacterial community composition in the formation water from 1.72 to 2.02 km of the Illinois Basin was characterized by analyzing 16S rRNA gene clone libraries. In order to evaluate the introduction of “contaminating” populations from the residual synthetic brine solution or drilling mud, the bacterial communities from both the partially contaminated intermediate and final samples from both depths were analyzed. Each clone library produced 136 to 149 high quality 16S rRNA genes (Table S2). Taxonomic analyses of the 16S rRNA genes showed similar microbial composition at phylum and class levels for all the four samples. The sequences were classified into Phyla Bacteroidetes, Firmicutes, Proteobacteria, SM2F11, and unclassified, with Firmicutes dominating all samples (Figure 3A). Within Phylum Firmicutes, Class Halanaerobiales OTUs dominated in all the samples, followed by Lactobacillales, Bacillales in samples 5655-S6 and 5655-S14, and Clostridiales in 5655-S14, 6634-S24, and 6634-S46 (Figure 3B).

An analysis of similarity (ANOSIM) of the community composition at the genus level suggested that their compositions were significantly different between the two source stratigraphic horizons (*P* < 0.001); while these differences were not observed between the intermediate and final samples within the same horizon. The number of OTUs identified using 97% as the cutoff ranged from 16 to 24 in the four samples (Table S2). Rarefaction analyses suggested that among the four samples, 5655-S14 was the only one suggesting that the clones were representative of the entire community composition (Figure S2). Higher numbers of OTUs were identified in the partially contaminated intermediate samples (5655-S6) compared to the final counterparts (5655-S14) (Figure 3C), suggesting a more diversified microbial community as would be expected. In contrast, similar OTUs were identified in the intermediate sample 6634-S24 and the final “clean” sample 6634-S42 (Table S2). Eight and seven OTUs occurred in both intermediate and final samples collected from 1.72 to 2.02 km, respectively (Figure 3C). The dominant Family *Halanaerobiales* occurred in all the samples with most of the OTUs affiliated with the Genus *Orenia* and the sequences most similar to those found in *Orenia marismortui* and *Orenia salinaria* (Moune et al., 2000) (Figure 3C).

### Enrichment culture experimentation

In order to evaluate whether the enriched bacteria could tolerate indigenous geochemical conditions, the enrichment cultures were transferred to fresh media where geochemical factors were varied; including temperature, salinity, pH, electron donors and alternative electron acceptors (e.g., iron-oxide minerals). Results indicated that both enrichment cultures catalyzed microbial iron reduction of ferrihydrite. Thus, all electron donor evaluations were performed using this poorly crystalized iron mineral as the electron acceptor.

**Figure 4:**
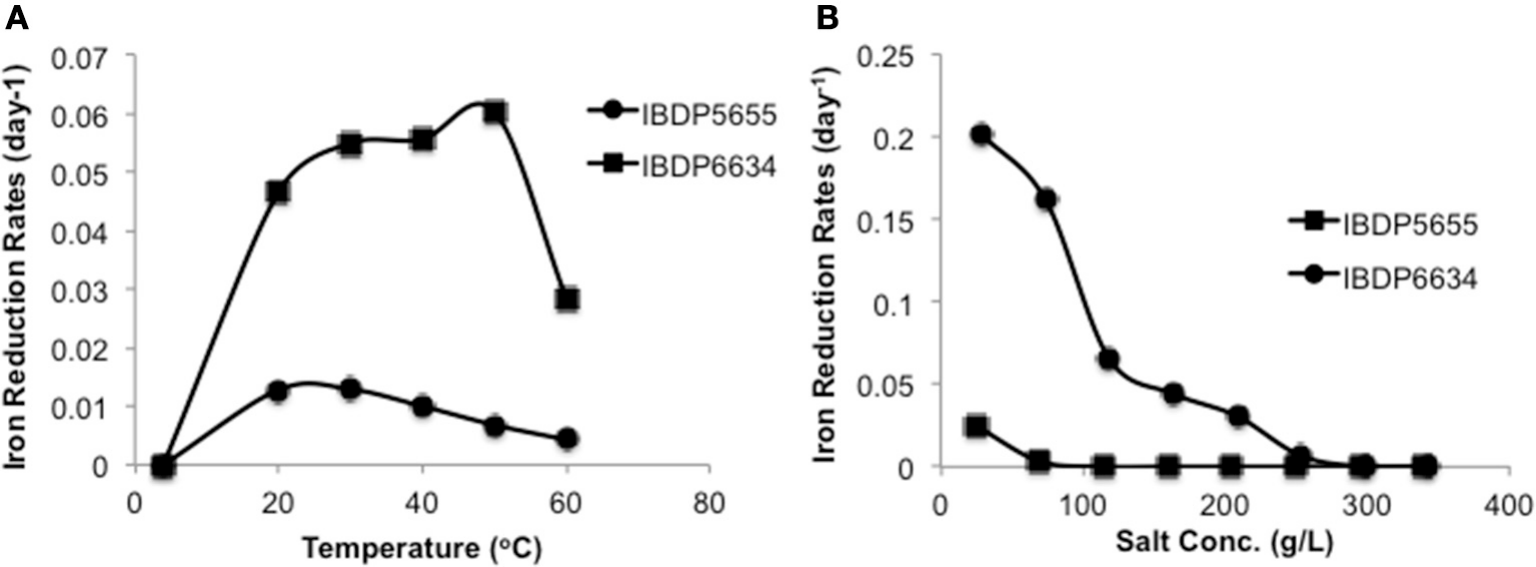
**Reduction of ferrihydrite by IBDP5655 and IBDP6634 at different temperatures (A) and salinities (B)**. The cultures for temperature tolerance **(A)** were prepared in the medium containing 40% (v:v) filter sterilized formation water. The cultures for salinity tolerance were grown at 42°C **(B)**. For both experiments, the un-inoculated controls were prepared for each condition and none of them showed visible iron reduction and are thus not included. Salt concentrations shown as percentage were calculated as the actual values containing the portion from basal medium, formation water and amended NaCl. Plots presented are average of duplicate samples at individual condition and the error bars are too small to be represented at the scale shown.

With ferrihydrite and at pH 7, ferric iron reduction occurred at significantly slower rates over a temperature range from 20 to 60°C with IBDP5655 (0.0043–0.013 day^−1^) compared to IBDP6634 (0.029–0.060 day^−1^) (Figure 4A). The optimal iron reduction activity for IBDP5655 may occur between 20 and 30°C, while for IBDP6634 first-order reaction rates were fairly consistent from 30 to 40°C with a slightly higher rate at 50°C. For both cultures, the iron reduction activity was significantly decreased at 60°C compared to that at lower temperature.

Gradual decreases in iron reduction rates were observed in response to increased salt concentrations for both enrichment cultures (Figure 4B). Both cultures exhibited the highest iron reduction rates in the presence of the lowest tested salt concentrations (10 g/L NaCl). A long lag time occurred prior to the onset of iron-reduction for IBDP5655 when the NaCl concentration was approximately 69 g/L and at further increased salt concentration, iron reduction activity in this culture was below detection limit. In contrast, measurable iron reduction rates were monitored in the IBDP6634 culture when NaCl concentration was as high as 200 g/L (Figure 4B).

In order to determine which substrates in the mixture of electron donors used in the original enrichment cultures actually contributed to growth of microorganisms in cultures IBDP5655 and IBDP6634, growth and activity tests with individual fatty acids or H_2_ were conducted. All the individual substrate tested supported slow iron reduction by the IBDP5655 culture. In contrast, IBDP6634 only utilized H_2_ and pyruvate as its electron donors (Figure S3). In addition, H_2_ supported significantly faster iron reduction activity and a greater extent of iron reduction than pyruvate (Figure S3).

The ability of each enrichment culture to use alternative electron acceptor and growth substrates was also evaluated. Other than ferric-iron reduction, both cultures exhibited fast fermentative growth in the presence of glucose. Enrichment culture IBDP5655 also grew with nitrate as an electron acceptor exhibiting stoichiometric production of nitrite with no further nitrite reduction observed. No nitrate reduction was observed in the IBDP6634 culture. Sulfate reduction and methanogenesis were not detected in either enrichment culture.

**Figure 5:**
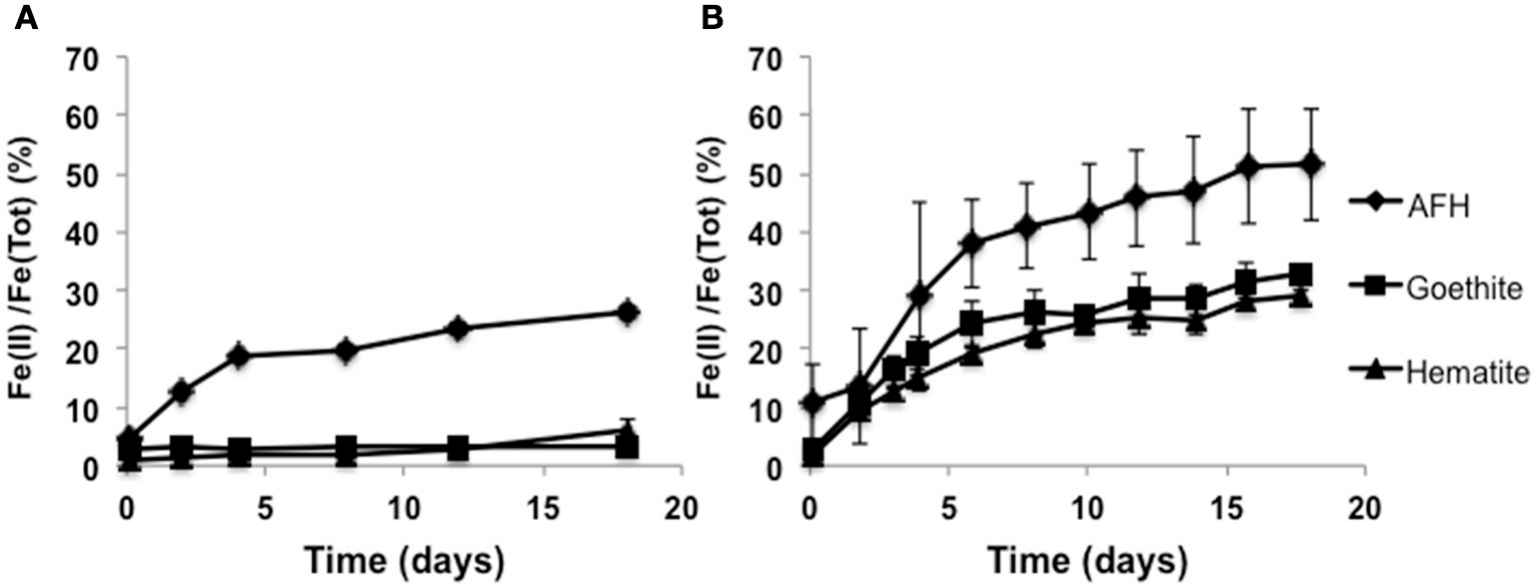
**Microbial iron reduction by the enrichment cultures IBDP5655 (A) and IBDP6634 (B) in the presence of different iron minerals**. pH of the cultures was approximately 6.2. All the samples were prepared in duplicate and the error bars indicate standard deviation of the replicates. Abiotic controls were prepared for all the conditions but without cell inoculation. No statistical changes in ferrous iron concentrations were observed in the abiotic controls (data not shown).

In order to evaluate the enrichment cultures' capacity to reduce more crystalline ferric minerals, IBDP5655 and IBDP6634 were grown in the presence of synthesized hematite or goethite. Both these minerals have been identified as coatings encrusting quartz crystals in the Mt. Simon Sandstone (Bowen et al., 2010). Both IBDP5655 and IBDP6634 actively reduced ferrihydrite with approximately 74 and 49% remaining after about 17 days of incubation, respectively (Figure 5). Enrichment culture IBDP6634 actively reduced both hematite and goethite (29–32%), however IBDP5655 showed a very poor ability to reduce these iron oxide minerals, with 6.2 and 3.1% reduction of hematite and goethite, respectively (Figure 5). For the same molar concentration, hematite contains twice as much iron as ferrihydrite. Thus, the actual total reduced iron with the hematite reduction by IBDP6634 was comparable to that produced during ferrihydrite reduction.

### FeO substrates reduction within enrichment cultures

**Figure 6:**
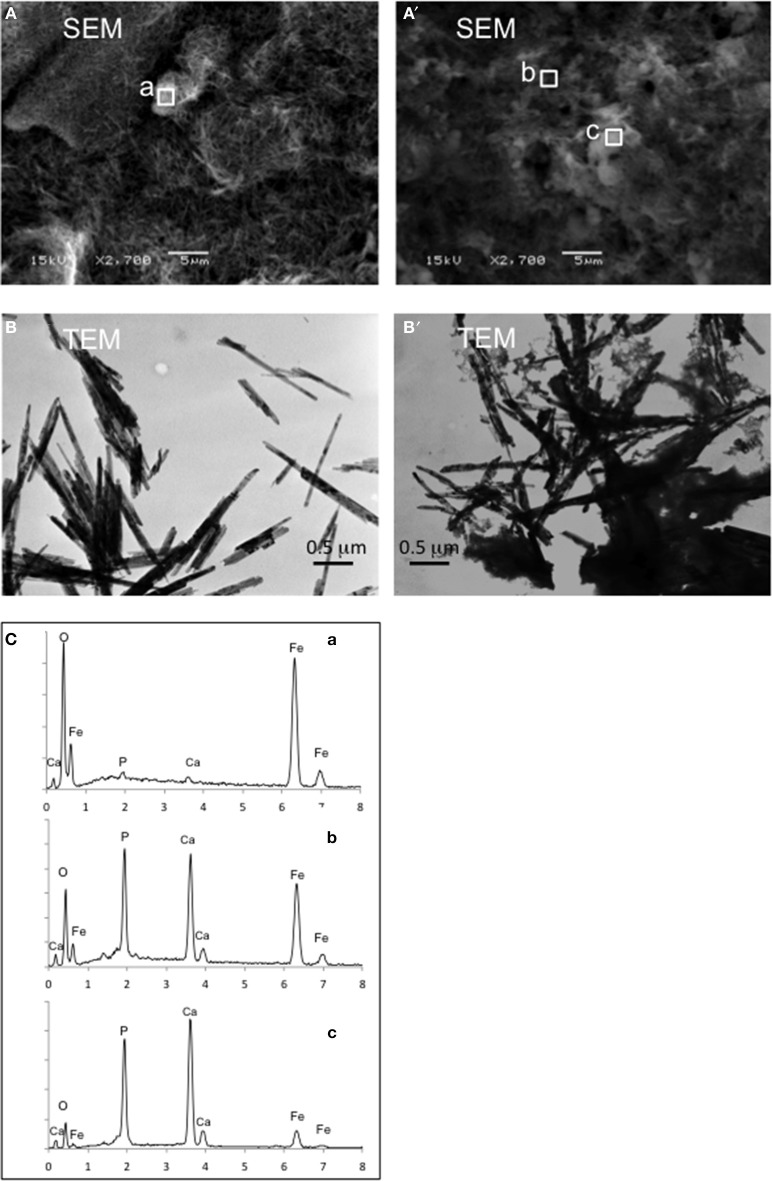
**Mineral morphology and element composition identified by SEM, TEM and SEM-EDX for goethite reduction by IBDP6634**. The control samples prepared and incubated under the same condition but without cell inoculation are illustrated as **(A,B)**; **(A')** and **(B')** show the mineral after being reduced by IBDP6634. Elemental compositions of the regions a, b, and c are shown in **(C)**.

SEM and TEM imaging show that changes in mineral crystal morphology were significant after iron reduction. For example, goethite lost its original well-crystalized needle shape after reductive dissolution by the IBDP6634 culture (Figure 6). This observation was further supported with the TEM images (Figure 6). Although semi-quantification of elements using SEM-EDX was not possible due to the porous mineral structure, analysis with this technique exhibited significant changes in elemental composition after iron reduction had occurred. Compared to the cell-free control that exhibited strong peaks for iron and oxygen, the peaks for calcium, phosphorous and sulfur became more significant in the samples incubated with the iron-reducing bacteria (Figure 6C). The efforts to determine the biogenic minerals in the iron-reducing cultures were not successful using XRD, which may be due to the poor crystal nature of any product generated or biogenic iron minerals in the concentrations lower than the detection limits.

## Discussion

The Mt. Simon Sandstone of the Illinois Basin exhibits stratigraphic and geochemical heterogeneity that may provide driving forces for abundant and metabolically diversified microbial life within this geological formation. Our previous study on the 1.8 km deep subsurface from the IBDP injection well (CCS1), Decatur, IL of the Illinois Basin provides the first snapshot of the microbial community inhabiting the Mt. Simon Sandstone, Decatur, IL. Metagenomic analyses of this microbial community in the context of indigenous physical, geological and geochemical factors suggest adaptive strategies by the dominant *Halomonas* species. For example, they may use nitrate, sulfate or organic matter from the environment and recycle intracellular and extracellular components to survive the oligotrophic habitat (Dong et al., 2014). The present study expands this previous investigation on understanding microbiota inhabiting different horizons of the Mt. Simon Sandstone. Here, the use of culture-dependent and -independent analyses demonstrates that this geological formation, with heterogeneous physical and geochemical conditions, harbors diverse microbial communities. These bacteria are stratigraphically differentiated but capable of sustaining reduction of the ferric iron minerals as one of the most abundantly known electron acceptors that are widely distributed on quartz grains in the Mt. Simon Sandstone (Bowen et al., 2010).

### Evaluation of contamination

Introduction of contamination from the drilling and well construction process is common for collecting deep subsurface samples (Pedersen, 1997). Therefore, in addition to the sampling strategies to collect representative formation water samples after extended purging and use a suite of geochemical parameters as “natural tracers,” we also used the two samples containing different extents of contaminants to evaluate contamination. Our analyses of the intermediate samples (5655-S6 and 6634-S24), collected prior to geochemical factors reaching stable conditions, reveal similar bacterial structure (*P* > 0.05) with their corresponding “clean” samples (i.e., 5655-S14 and 6634-S46). Using qPCR targeting the predominant OTUs enriched from each “clean” sample shows, however, that the specific populations (OTUs Z9S14I64 for 5655-S14 and Z6S24I11 for 6634-S46) exhibit significantly higher abundance in the source samples compared with the intermediate ones, 5655-S6 and 6634-S24 (Figure 3). This supports the premise that these are resident populations to the formation. Some phylotypes in the contaminated samples (e.g., clones Z9S6I42, Z9S6I23 and Z6S24II3) are closely related to bacterial species from the environments significantly different from the deep subsurface ecosystem (i.e., fresh water aquifer, soil, and tidal flats) (Shaw et al., 2008; Filippini et al., 2011). Since these apparent drilling fluid associated phylotypes occur only in the partially contaminated samples and they represent a low fraction of the total bacterial community at each sampling depth, it is likely that the majority of the recovered phylotypes in the “clean” samples are representative of the indigenous biosphere.

### The deep subsurface biosphere of the Mt. Simon sandstone in samples 5655-S14 and 6634-S46

Both of the subsurface formation water samples collected in the present study from the Mt. Simon Formation (1.72 and 2.02 km) contain low diversity bacterial communities dominated by Halanaerobiales, a member of Phylum Firmicutes. These community characteristics are not unusual in the studies of terrestrial deep subsurface microbial consortia. Bacterial communities inhabiting > 1 km-deep granitic-fracture water in Henderson Mine, CO, contained OTUs primarily affiliated with Firmicutes, Proteobacteria and a new phylum (Sahl et al., 2008). Lin et al. (2006) reported a deep subsurface bacterial community dominated by a single phylotype related to thermophilic sulfate reducers in the Firmicutes sustained by sulfate and hydrogen for millions of years without photosynthetic inputs (Lin et al., 2006). The Mt. Simon Sandstone is also disconnected from recharge of surficial water containing photosynthesis-derived organic nutrients due to the multiple overlying low permeability shale layers (e.g., Eau Claire and New Albany Shales) (Strapoć et al., 2009; Panno et al., 2013). Thus, the microbial communities within the Mt. Simon Sandstone sustain themselves in the habitat that is energy-limited and only the populations that can adapt to the indigenous physical, geochemical and oligotrophic stresses can survive.

The microbial community composition of samples 5655-S14 and 6634-S46 reflects the geochemical features of their subsurface habitats present within the Cambrian-age Mt. Simon Sandstone, which includes mesothermal, saline, moderately pressurized and oligotrophic rock burial environments (Table 1). Therefore, the extreme thermophiles commonly found in the higher temperature subsurface environments (e.g., oil fields, mine or marine hydrothermal vent sediments) (Fredrickson and Balkwill, 2006) were not detected in this work. The identified 16S rRNA gene sequences (phylotypes), however, found in the “clean” samples (5655-S14 and 6634-S46) are phylogenetically related to mesophilic and halotolerant bacteria inhabiting comparable subsurface ecosystems. For example, a group of OTUs (e.g., Z6S24I14 and Z9S14I15) account for the majority of 16S rRNA gene clones found in the 5655-S14 and 6634-S46 bacterial communities (Figure 3). They share 97–99% sequence identity to 16S rRNA genes found in bacteria identified in a high-temperature and hypersaline North Sea oil-field (Dahle et al., 2008). Their closest phylogenetically related cultured organisms are a group of fermentative anaerobic halophilic bacteria isolated from saline environments (e.g., *Orenia marismortui* (Oren et al., 1987) from the Dead Sea, *O. salinaria* (Moune et al., 2000) from sediments of a Mediterranean saltern and *O. sivashensis* from a salty lagoon (Zhilina et al., 1999). These strains show 92–96% identity in 16S rRNA genes with the *Orenia*-related OTUs found in DNA extracted from samples 5655-S9 and 6634-S46. Additionally, clone Z9S6I38 is closely related to *Halolactibacillus miurensis* and *H. halophilus*, the marine-inhabiting lactic acid bacteria that survive salt concentration up to 25% (Ishikawa et al., 2005). Other OTUs, such as clones Z9S6I30 and Z9S6II76, are phylogenetically close to *Vulcanibacillus modesticaldus* (L'Haridon et al., 2006) that is capable of growing anaerobically up to 60°C (Gray and Engel, 2013). The detection of phylotypes related to those adapted to elevated temperatures and salt concentrations suggest that they are indigenous to the Mt. Simon Sandstone samples from the VW1 at 1.72 and 2.02 km below surface.

### Heterogeneity of subsurface bacterial communities within the Mt. Simon sandstone

Interestingly, a sample from a similar depth in a nearby bore hole in the Mount Simon Sandstone (Dong et al., 2014) yielded a bacterial community significantly different from those found in 5655-S9 and 6634-S46. This bacterial consortium was found in our previous work at 1.8 km below the surface from the CCS1 (referred to as 5872 afterwards), which is about 300 m due south of VW1 in the present study. More than 97% of the analyzed 16S rRNA gene sequences in this sample are affiliated with *Halomonas*, a genus within γ-Proteobacteria (Dong et al., 2014). In contrast, although horizons 5655 and 6634 are separated vertically by about 300 meters and show some bacterial community differences, both are dominated by Firmicutes (Figure 3).

The differences among the bacterial communities of the Mt. Simon Sandstone pore waters between the Decatur monitoring well (VW1; this study) and injection well (CCS1) (Dong et al., 2014) suggest spatial heterogeneity and complexity of physical, geochemical and microbial niches within this geological formation. The Mt. Simon Sandstone is extremely laterally and vertically heterogeneous with respect to depositional rock types and their associated mineralogy, porosities and permeability (Bowen et al., 2010). As a result, significant changes can occur in pore water chemistry and associated bacterial community composition, even over short vertical and lateral differences (i.e., 300 m between VW1 and CCS1). The physical and geochemical parameters in the present work (Table 1) and our previous study (Bowen et al., 2010) are consistent with studies that demonstrate stratified halide and halide-cation ratios within the Mt. Simon Sandstone of Illinois Basin (Bowen et al., 2010; Panno et al., 2013). Although movement of brines out of Mt. Simon Sandstone and/or exchange of brines with other formations are constrained, the formation water within this formation have been under various extents of mixing of the original saline groundwater with other water source (e.g., ore-forming brines, seawater, seawater evaporated short of halite saturation or other groundwater) (Panno et al., 2013), which may also lead to introduction of bacterial communities from different sources. These are analogous to the vertical changes of a 2.3 km deep crystalline rock of the Fennoscandian shield, Finland, and the horizontal gradient observed in deep-granitic-fracture water in Colorado, US. In both studies, significant bacterial composition diversity was observed in response to varied source of drill hole water and formation mechanisms in addition to their local geochemistry (Sahl et al., 2008; Nyyssonen et al., 2014).

Heterogeneous geochemical and microbial niches within the Mt. Simon Sandstone may derive from the significant depositional (primary) and post-depositional (secondary) alternation processes (Miller, 2011) of this geological formation. The Mt. Simon Sandstone overlays a Precambrian granite and rhyolite basement (Sloss, 1963; Kolata and Nelson, 1991; Kolata, 2010) and extends to the overlaying upper Eau Claire Shale (Strapoć et al., 2009; Panno et al., 2013). Sediment deposition derives from high hydrologic energy environment dominated by terrestrial alluvial fans to braided river deposition on shallow marine shelf environments (Boggs, 2001; Leetaru and McBride, 2009; Leetaru et al., 2009) at the bottom intervals to the terrestrial to shallow-water marginal marine environments (Driese et al., 1981; Leetaru et al., 2009; Bowen et al., 2010) at the shallower ones. This deposition history is reflected by the petrographic studies showing transition of sediment particle morphology from moderate to well-sorted quartz arenite to similar arenite interbedded with fine-grained shale layers, increased porosity and permeability from lower to the upper stratigraphic intervals of the Mt. Simon Sandstone (Bowen et al., 2010; Miller, 2011). This complexity in deposition, post-depositional alteration processes, and resulting geochemistry of the Mt. Simon Sandstone all possibly influence introduction, habitation, adaptation, and distribution of different microbial communities within the Mt. Simon Sandstone.

### Active iron-reducing enrichment cultures

Although the *Vulcanibacillus*- and *Orenia*-related species were identified in the bacterial communities derived from both VW1 sampling horizons (Figure 3), they were not equally enriched in the cultures. The selective enrichment of one over the other might be due to the distinct physiological properties of the two major groups. Enrichment IBDP6634, containing *Orenia*-related species, exhibits significantly higher tolerance to changes in salinity and temperature (Figure 4). Thus, the *Orenia* population in enrichment cultures IBDP6634, with a relatively high salt concentration (approximately 83 g/L TDS), may outcompete the *Vulcanibacillus*-related species. Meanwhile, a higher capacity to reduce dissolved ferric citrate (Figure S2) and a variety of fatty acids (Figure S3) might enable the *Vulcanibacillus*-related populations to be more active compared to the *Orenia*-related species when the salt concentration was feasible for their growth.

It is important to validate that *Vulcanibacillus* and *Orenia* populations are indigenous (Figure 2) and representative of a iron-reduction driven ecosystem. Phylogenetically diverse Bacteria and Archaea actively carry out respiratory iron reduction and have been obtained from geographically and geochemically diversified environments (Weber et al., 2006). No iron-reducing capacity, however, has been reported for *Vulcanibacillus* and *Orenia* spp, which have been typically studied as nitrate reducers and fermenters, respectively (Zhilina et al., 1999; Moune et al., 2000; L'Haridon et al., 2006). Since the 16S rRNA gene sequence identity for the enrichment populations ranges from 95 to 96% compared to their closest phylogenetic relatives (e.g., *V. modesticaldus*, *O. marismortui* and *O. salinaria*), it is not known whether these enriched populations are affiliated to new genera or belong to *Vulcanibacillus* and *Orenia* but with novel or unexplored physiological properties. Further physiological characterization of the isolated cultures from these enrichment cultures is underway.

The iron-reducing capacity by the *Vulcanibacillus* population in Enrichment 5655 shows similarity with most previously published iron-reducing organism physiology studies in that they actively reduce only dissolved and poorly crystalline ferric iron (e.g., chelated Fe(III) or ferrihydrite) (Lovley, 1991; Lovley et al., 2004). In contrast, the *Orenia*-populated enrichment exhibits exceptional iron-reducing capacity for both poorly crystalline ferrihydrite and well-structured crystalline hematite and goethite. So far, only a handful of iron reducers have been found to be capable of deriving energy from active reduction of poorly crystalline hematite and goethite, such as *Geobacter sulfuroreducens*, *G. metallireducens*, *Shewanella purtrefaciens* strain CN32, *S. oneidensis* MR-1 and *S. alga* strain BrY (Roden and Zachara, 1996; Roden and Urrutia, 1999; Royer et al., 2002, 2004; Yan et al., 2008; Bose et al., 2009). Investigation of the enzymatic mechanisms by these iron-reducing organisms indicates they employ a variety of strategies, such as direct contact on solid iron minerals (Nevin and Lovley, 2000; Reguera et al., 2005), synthesis of pili as nanowire conduits for electron transfer (Reguera et al., 2005) or exogenously produce electron mediators (Lovley et al., 1996; Newman and Kolter, 2000), to overcome thermodynamic favorability of the well-structured crystalline ferric iron minerals and live “on the energetic edge” (Weber et al., 2006).

The iron-reducing populations found in IBDP5655 and IBDP6634 are affiliated to Firmicutes, the group that has been relatively poorly understood in their iron reduction mechanisms. The genomes of several Firmicutes have abundance of multi-heme-cytochrome genes (Sharma et al., 2010). Limited knowledge about a Gram-positive Firmicutes, *Carboxydothermus ferrireducens*, shows expression of c-type cytochromes during metal reduction (Gavrilov et al., 2007). However, it is not well understood whether they employ similar mechanisms as the well understood representative Proteobacteria (e.g., *Geobacter* spp and *Shewanella* spp) who transfer electron from cytoplasm to the extracellular solid phase electron acceptors via a series of (meta) quinones and cytochromes with overlapped midpoint potentials (Weber et al., 2006; Bird et al., 2011; Richardson et al., 2012; Shi et al., 2012).

### Implications for predicting indigenous subsurface microbial communities

High concentration of dissolved ferrous iron in the pore water of Mt. Simon Sandstones may derive from multiple sources. Physiological characterization of the enrichment cultures in the present study suggests that ferrous iron can be generated from microbial iron reduction. Alternatively, it may derive from introduced marine water or leached from anaerobic soil from the Archean Era (Amy and Haldeman, 1997) and have been buried since this geological formation had been deposited. Although it is not possible to determine the relative contribution of either process, the ubiquity of ferric iron minerals, presence of active iron-reducing organisms capable of reducing indigenous iron oxides (e.g., hematite and goethite) and detection of H_2_ (a potential electron donor) during subsequent sampling of VW1 from the same zones for this study (ISGS, personal communication) imply that the factors needed for microbial iron reduction can be met in the two VW1 horizons sampled.

It is speculated that iron cycling within the two VW1 horizons of Mt. Simon Sandstone might be sustainable processes. Along the burial history of this geological formation, introduction of oxygen-containing meteoric water (e.g., rainfall) (Lewy, 2013) or incident radiation (Braterman et al., 1983) may repeatedly precipitate ferric iron (e.g., hematite and goethite), which is supported by the petrographic relationships observed in many deep subsurface sediment samples from Mt. Simon Sandstone of the Illinois Basin (Bowen et al., 2010). The precipitated ferric oxides may act as an alternative electron acceptor for survival and growth of iron-reducing organisms, coupling organic or inorganic compounds (e.g., H_2_) as the electron donor(s). Due to the weakly acidic condition of the groundwater, most reduced Fe(II) may stay in the aqueous phase (Figure S4) and thus accumulates the relatively high soluble Fe(II) concentration. Although it is not known when the iron-reducing organisms were introduced, the description of meteoric water introduction (Stueber et al., 1987; Stueber and Walter, 1993) and the relatively hydraulically disconnected nature of the Mt. Simon Sandstone suggests that some indigenous microorganisms have evolved microbial iron reduction within this isolated environment.

The differential physiological properties of the two enrichment cultures in this study (e.g., tolerance of temperature, salinity and ability to reduce different ferric iron minerals) suggest their type of metabolism in the indigenous ecosystem may vary significantly. For example, the *Vulcanibacillus*-related populations did not grow or exhibit activity under culture conditions that simulated the natural ecosystem (e.g., high temperature, salinity and presence of well-structured crystalline ferric iron minerals) (Figures 4, 5), thus these organisms may be dormant compared to the *Orenia*-related populations which are very active under these same simulated conditions.

## Conclusion

Geochemical data, phylogenetic analyses of the bacterial communities and cultivation of iron-reducing bacteria (novel genera or unexplored physiological properties of Genera *Vulcanibacillus* and *Orenia*) from 1.72 to 2.02 km horizons of the Mt. Simon Sandstone of the Illinois Basin, IL, suggest interactions between indigenous environmental conditions and the bacterial communities. For instance, bacterially mediated iron reduction may sustain the turnover of carbon and/or H_2_ in this ecosystem.

This study provides important microbial baseline information for the IBDP. Although injection of super-critical phase CO_2_ will certainly alter the viability and composition of the indigenous microbial communities (Wu et al., 2010), some studies suggest adaptation of microbial communities to the changes and return to the pre-injection composition over time (Morozova et al., 2010). Establishing a baseline of microbial communities is important for understanding the long-term biogeochemistry of the CO_2_ storage reservoirs for the potential interaction of CO_2_ with the indigenous geochemical factors mediated by microbial metabolisms. In our study, high concentrations of dissolved Fe(II), abundance of ferric iron minerals (Bowen et al., 2010) and prevalent iron-reducing microbial communities suggests that potential precipitation of carbonate by Fe(II) to form amorphous iron(II) carbonate as a precursor for siderite (FeCO_3_) (Sel et al., 2012), balancing pH decrease by dissolution of indigenous ferric iron minerals or microbial iron reduction (Onstott, 2005) may provide natural enhancement for CO_2_ storage.

Further studies are focused on characterization of the *Vulcanibacillus*- and *Orenia*-related isolated pure cultures to understand their comprehensive physiological characteristics. Genomes of these novel iron-reducers have been sequenced. Genome reconstruction, together with physiology studies will facilitate understanding of the metabolic strategies of these novel iron-reducing organisms.

### Conflict of interest statement

The authors declare that the research was conducted in the absence of any commercial or financial relationships that could be construed as a potential conflict of interest.
