# Supplementary material for: Fe-oxide grain coatings support bacterial Fe-reducing metabolisms in 1.7−2.0 km-deep subsurface quartz arenite sandstone reservoirs of the Illinois Basin (USA)
Source: Front Microbiol. 2014 Sep 30;5:511. doi: 10.3389/fmicb.2014.00511 (PMC4179719; doi:10.3389/fmicb.2014.00511)
Supplement: Supplementary file 1 [file Presentation1.PDF]

Supplementary Information for

Fe-oxide grain coatings support bacterial Fe-reducing metabolisms in 1.7-2.0 km-deep subsurface quartz arenite sandstone reservoirs of the Illinois Basin (USA)

Yiran Dong[1,2,3]*, Robert A. Sanford[2], Randall A. Locke II[4], Isaac K. Cann[1,3,5,6], Roderick I. Mackie[1,3,5], Bruce W. Fouke[1,2,3,4,6]

Institute for Genomic Biology, University of Illinois, Urbana-Champaign[1]
Department of Geology, University of Illinois, Urbana-Champaign[2]
Energy Biosciences Institute, University of Illinois, Urbana-Champaign[3]
Illinois State Geological Survey, Urbana-Champaign[4]
Department of Animal Sciences, University of Illinois, Urbana-Champaign[5]
Department of Microbiology, University of Illinois, Urbana-Champaign[6]

*Correspondence: Dr. Yiran Dong, Institute for Genomic Biology, University of Illinois, Urbana-Champaign, 1206 W. Gregory Drive, Room 3500, Urbana, IL, 61801, USA.
dong5600@illinois.edu

S1. Methods and material

S1.1. Sample collection and treatment

The two formation water samples collected from each horizon were used for characterization of resident microbial communities. Unless mentioned, about 14 liters of each sample was collected in sterile 1 L Nalgene® bottles and 10 L polypropylene (PP) carboys (Thermo Scientific, MA). All the containers were sterilized in the laboratory at UIUC by sequential wash with soap water, freshly prepared bleach solution (10%) and then nanopure water rinse for at least 5 times before autoclaving. Restrictive sterilization was followed in order to avoid/minimize potential contamination by mud, dirt or residual DNA fragments.

The sample bottles were immediately transported to the on-site mobile laboratory, put on ice, and split into different several subset volumes for subsequent geochemical and microbiological analyses. These included: (1) two 40 mL glass EPA vials (Thermo Fisher Scientific, PA) that were acidified with 1 mL 5 N HCl to final pH of less than 3 for total organic carbon (TOC), ferrous iron and ion measurements; (2) a Nalgene® plastic bottle with minimal headspace and sealed with electrical tape for enrichment cultures and bacterial cell enumeration; and (3) filtration of the remaining fluid through sterile PES membrane filters (0.22 μm in pore size, Thermo Scientific, PA) into 1 L Nalgene bottles. The filter membrane was cut with a sterile blade and immediately transferred into sterile 50 mL conical tubes containing 10 mL RNAlater (Applied Biosystem/Ambion Inc., TX). All the samples were then placed on ice in a cooler and shipped back to the laboratory at the end of each sampling day and stored in dark at 4 °C. The conical tubes containing membranes and RNAlater were transferred to an -80 °C degree freezer for long-term storage after they were incubated at 4 °C for one day following the manufacturer's instruction.

S1.2. Geochemical analyses

A full suite of geochemical parameters was measured at the wellhead or field laboratory by ISGS staff. Parameters, including pH, temperature, dissolved oxygen, specific conductance and Eh, were measured with an ORION® 5-Star pH/RDO*/Conductivity portable meters (Thermo Scientific, MA) and associated probes. Density of formation water was measured with a Mettler-Toledo Densito 30PX portable density meter (Mettler-Toledo Inc., OH). Concentration of potassium was semi-quantified with QUANTOFIX potassium test strips (Macherey-Nagel Inc., PA). Ammonia and ammonium were measured with JT Baker Test strips (Mallinckrodt Baker Inc., NJ) and HACH Water Quality Test Strips (HACH Company, CO), respectively, following the manufacturer's instruction.

Additional geochemical analyses for iron species, total dissolved solids (TDS), total nitrogen (TN) and TOC were completed in the laboratory at the UIUC. Ferrous iron was measured in the acidified sample splits. In order to avoid exposure to the atmosphere, the formation water for swab sampling was not filtered through membrane before acidification. The concentration of ferrous iron identified (defined as $Fe(II)_{tot}$) includes both dissolved Fe(II) and surface associated Fe(II) extracted from the small proportion of sedimentary particles (< 1%, v/v). $Fe(II)_{tot}$ was measured with ferrozine methods (1, 2) using a Genesys 20 spectrophotometer (ThermoFisher Scientific Inc., MA) at 562 nm. The samples were diluted in 0.5 N HCl if the concentrations were beyond the range of standards.

TDS, TN and TOC were measured using 20 ml subsamples of formation water after filtration through a 0.2 μm syringe filter (Thermo Fisher Scientific, PA). The filtrate for TDS analysis was freeze-dried by using a LABCONCO Freeze Dry System/Freezone™ 4.5 (Labconco Corporation, MO) and identified using a flash combustion chromatographic separation elemental analyzer (Costech 4010 CHNSO Analyzer, Costech Analytical Technologies Inc. Valencia, California, USA). TOC was measured with the filtered formation water using a Shimadzu TOC analyzer 5000 (Shimadzu Inc., Japan). The

liquid was pretreated with 0.2 μm syringe filters (Thermo Fisher Scientific, PA) and 5 N HCl as described above and stored at 4 °C before analysis.

S1.3. Physiological properties of iron-reducing enrichment cultures

In order to evaluate whether the enrichment cultures could tolerate the indigenous environment in the subsurface, a series of growth/activity assays with simulated geochemical parameters, including temperature, salt concentration and nutrients (e.g., electron acceptors and donors) were prepared. Unless mentioned, all the cultures were prepared in the basal medium amended with 10% (v/v) of subsurface formation fluid. Except for the experiment to understand impacts of temperature on microbial activity, all the cultures were incubated at 42 °C in the dark and manually shaken every day.

To evaluate impact of temperature on microbial activity, cultures were incubated at the temperatures ranging from 20 to 60 ºC at 10 ºC intervals in the water baths. Effect of salinity was assessed by amending culture tubes with different concentrations of NaCl ranging from approximately 1% to 35% by mass. Iron reduction rates were calculated as first order constants assuming the metabolic reactions depended on concentrations of ferric iron minerals (3). Contribution of the electron donors was assessed for the components of electron donor mixture, including acetate, lactate, pyruvate and formate, by incubating one of them (5 mM) with iron-reducers in the presence of ferrihydrite (5 mM) as the electron acceptor. Electron donor utilization was assessed using iron-reducing activity as a positive indicator of use. For these experiments, microbial activity of was evaluated based on ferrous iron production rates in the cultures.

Four different alternative electron accepting processes, including nitrate-reducing, sulfate-reducing, fermentation and methanogenesis were evaluated in each enrichment culture. In the nitrate- and sulfate-reducing enrichment cultures, $NaNO_3$ and Na_2SO_4 (5 mM) were amended in the presence of the same electron donors (acetate, lactate, pyruvate, formate (5 mM each) and H_2 (5 mL/25 mL tube)). Ten mM of glucose was added to evaluate fermentation capability. For methanogenic conditions, 5 mM methanol was added to the media. Growth of the organisms was monitored by measuring the change in optical density (OD_{600}) using a SPECTRONIC 20D+ spectrophotometer (Thermos Scientific, MA).

Iron-reducing activity was evaluated beyond dissolved (e.g., ferric citrate) and poorly crystalline ferrihydrite, which were initially used in the enrichment cultures. Hematite [Fe_2O_3], goethite [α-FeO(OH)] and lepidocrocite [γ-FeO(OH)] were tested as the electron accepters in each electron acceptor. The produced ferrous iron was monitored over time in the cultures amended with approximately 10 mM of each iron-oxide mineral. Ferrihydrite, hematite, leptochrocite and goethite were synthesized following the published methods (4). These synthesized minerals were washed with Nanopure water for at least 6 times and resuspended in Nanopure water with a nominal final concentration of approximately 1 M. The mineral suspensions were bubbled under N_2 for at least 30 minutes before being sealed in serum bottles and autoclaved. Crystal structure of the synthesized minerals was confirmed with X-ray diffraction (XRD) at the Material Research Laboratory, UIUC.

S1.4. Morphological and mineralogical analyses

Mineral morphology and chemical composition were determined in iron-reducing enrichment cultures by observing prepped samples with both a scanning electron microscope (SEM) and a transmission electron microscope (TEM). In order to minimize re-oxidation of the reduced iron minerals, sample preparation for microscopic analyses was initiated inside an anaerobic chamber (Coy Laboratory Inc., MI) filled with N_2:H_2 mixture (95:5, v/v). Samples for SEM analysis were filtered through polycarbonate membrane (0.22 μm in pore size). The samples were promptly washed with anaerobic sterile PBS buffer (pH 7.4). The Pellets were air-dried

inside the anaerobic chamber and stored in an anaerobic container before transported to the microscopy laboratory.

Sample observation was under a JEOL JSM-6060LV Low Vacuum Scanning Electron Microscope (JEOL Ltd., Japan) connected with an Oxford Instruments ISIS EDS System 10 mm^2 ATW Si(Li) X-ray Detector 130 eV resolution (Mn K-alpha) (Oxford Instruments, UK). The samples for TEM analysis were prepared from the pellets after centrifuging well-mixed culture for at 10,000 rpm for about 3 minutes. Samples were observed under a HITACHI H600 Transmission Electron Microscope with H-6010 A Scanning System (HITACHI Co, Japan).

Secondary minerals formed in the enrichment culture were also analyzed using XRD analysis. The samples were prepared from centrifuged culture pellets. To avoid re-oxidation of reduced ferrous iron, the pellets were mixed with Vaseline and were evenly cover on slides. XRD analyses were performed using a Siemens/Bruker D5000 theta/theta XRD system (Siemens AG, Germany) (40 kV, 35 mV) equipped with Co-Kα radiation with a scan rate 2° 2θ /min. The results of XRD were analyzed with the aid of MDI Jade 6.1.

S2. Tables and figures

Table S1. qPCR primers used to quantify enriched iron-reducing bacteria and total bacterial 16S rRNA genes

Primer	Sequence	Target gene	Length (bp)	Reference(s)
Z9For	5'-AAGAGAACATCTCGCATGAG	IBDP5655	302	This study
Z9Rev	5'-GGCTTGTTCATCGGATACTT	16S rRNA		
Z6For	5'-CTTCTTCTTAGGTATCGCTT	IBDP6634	268	This study
Z6Rev	5'-GCTGGTATTAGCAACTAAAG	16S rRNA		
Bac1055YF	5'-ATGGYTGTCGTCAGCT	*Bacteria*	337	(5)
Bac1392R	5'-ACGGGCGGTGTGTAC			

Table S2. Summary of 16S rRNA gene clone libraries of the bacterial communities collected from 1.72 and 2.02 km of VW1, Mt. Simon Sandstone, Decatur, IL

	5655-S6	5655-S14	6634-S24	6634-S42
Number of sequences	138	141	145	149
Number of OTUs	31	18	17	20
Richness (Chao)	115	23	43	53

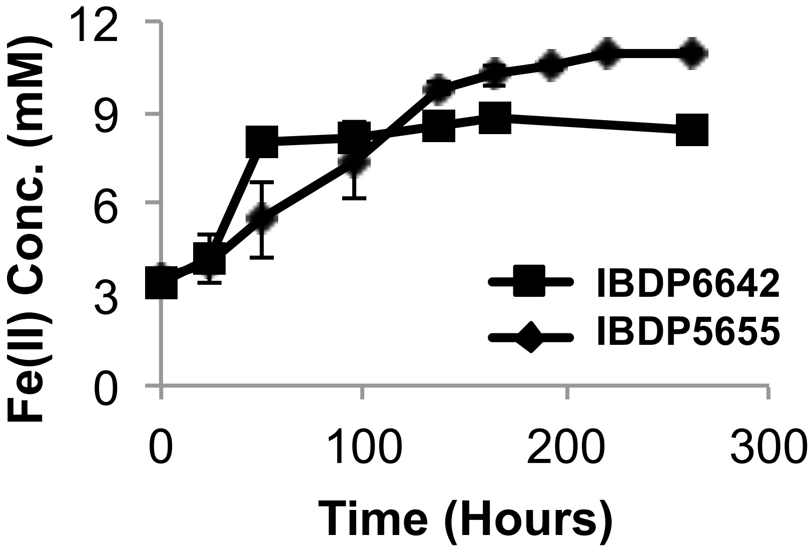

Figure S1. Reduction of ferric citrate (about 15 mM) by active enrichment cultures IBDP5655 and IBDP6634 fed with approximately 15 mM ferric citrate at 42 °C. Samples were prepared in replicates and the error bars denote standard deviation of duplicate samples. Some of the error bars are too small to be visualized. In the un-inoculated controls, no visible iron reduction was observed and is not shown.

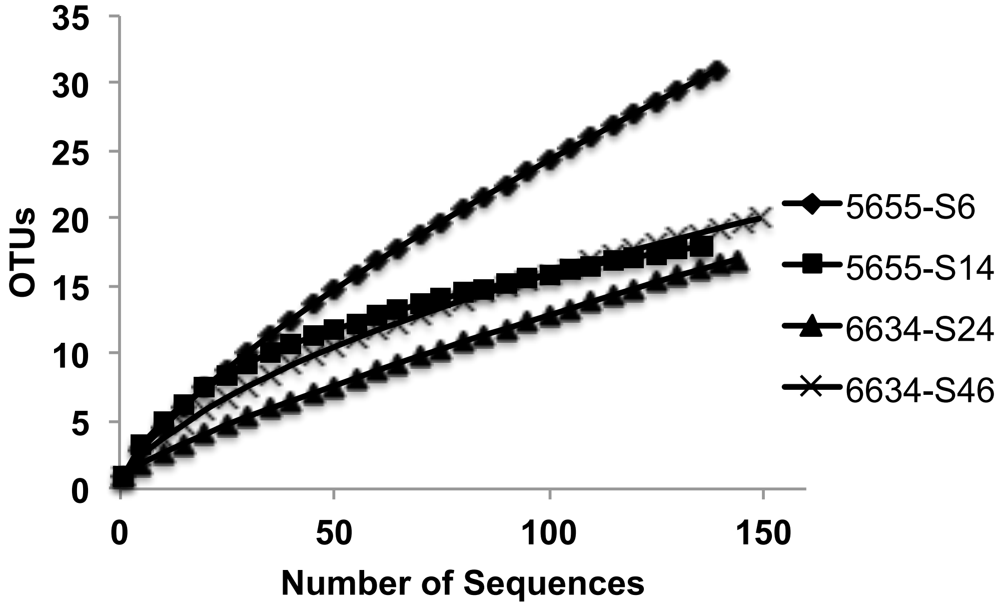

Figure S2. Rarefaction of bacterial communities identified in the formation water collected from 1.72 and 2.02 depth horizon in the well VW1 of the Mt. Simon Sandstone, Decatur, IL.

(A) (B)

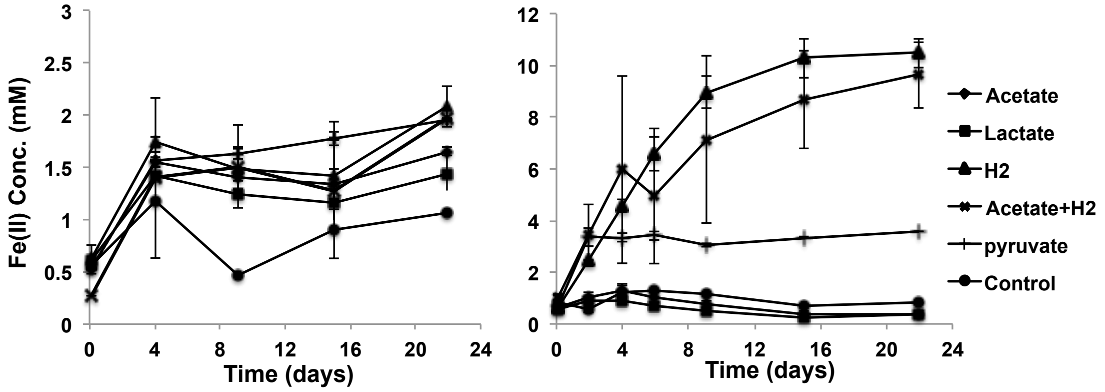

Figure S3. Bacterial iron reduction in the presence of different electron donors by IBDP5655 (A) and IBDP6634 (B). All the cultures prepared at pH 7.0 and amended with approximately 15 mM ferrihydrite. They were incubated statically at temperature 42 °C. Control means the samples inoculated with the same enrichment culture but in the absence of electron donors. The samples were prepared in duplicates and the error bars indicate standard deviation of replicates.

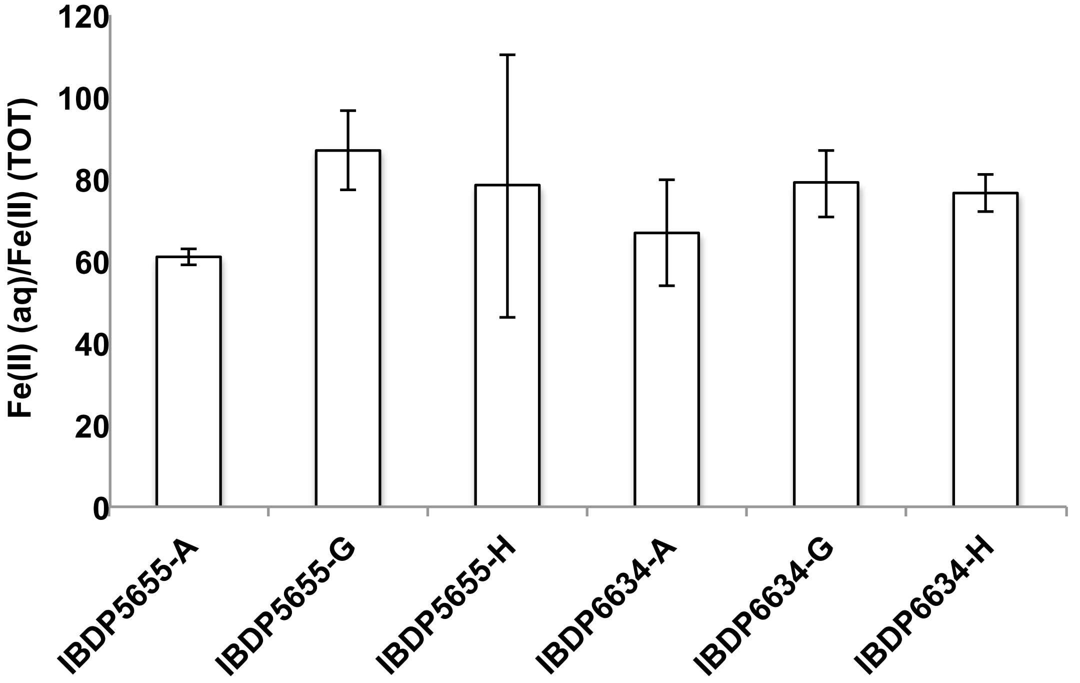

Figure S4. The majority of reduced ferrous iron existed as dissolved Fe(II) in the aqueous phase during reduction of ferrihydrite (A), goethite (G) and hematite (H) by the enrichment cultures IBDP5655 and IBDP6634. The columns show average values of duplicate samples under each condition and the error bars indicate standard deviation of the replicates.

S3. References

1. Gibbs, C. R.: Characterization and application of ferrozine iron reagent as a ferrous iron indicator. Analytical Chemistry, 48, 1197-1200 (1976)

2. Stookey, L. L.: Ferrozine - a new spectrophotometric reagent for iron. Analytical Chemistry, 42, 779 (1970)

3. Jensen, M. M., B. Thamdrup, S. Rysgaard, M. Holmer and H. Fossing: Rates and regulation of microbial iron reduction in sediments of the Baltic-North Sea transition. Biogeochemistry, 65(3), 295-317 (2003)

4. Schwetmann, U. and R. M. Cornell: Iron Oxides in the Laboratory: Preparation and Characterization. Wiley-VCH Verlag GmbH, Weinheim (2000)

5. Lane, D. J.: 16S/23S rRNA sequencing. In: Nucleic Acid Techniques in Bacterial Systematics. Ed E. Stackebrandt&M. Goodfellow. John Wiley & Sons, Chichester, United Kingdom (1991)
